# Supplementary material for: The interactome of intact mitochondria by cross-linking mass spectrometry provides evidence for coexisting respiratory supercomplexes
Source: Mol Cell Proteomics. 2017 Dec 8;17(2):216–32. doi: 10.1074/mcp.RA117.000470 (PMC5795388; doi:10.1074/mcp.RA117.000470)
Supplement: Supplemental Data [file supp_RA117.000470_133922_0_supp_23978_fzffwf.zip › spectra_annotation/mito_DR_spectra_annotation/428-1-1-1-1-1.pdf]

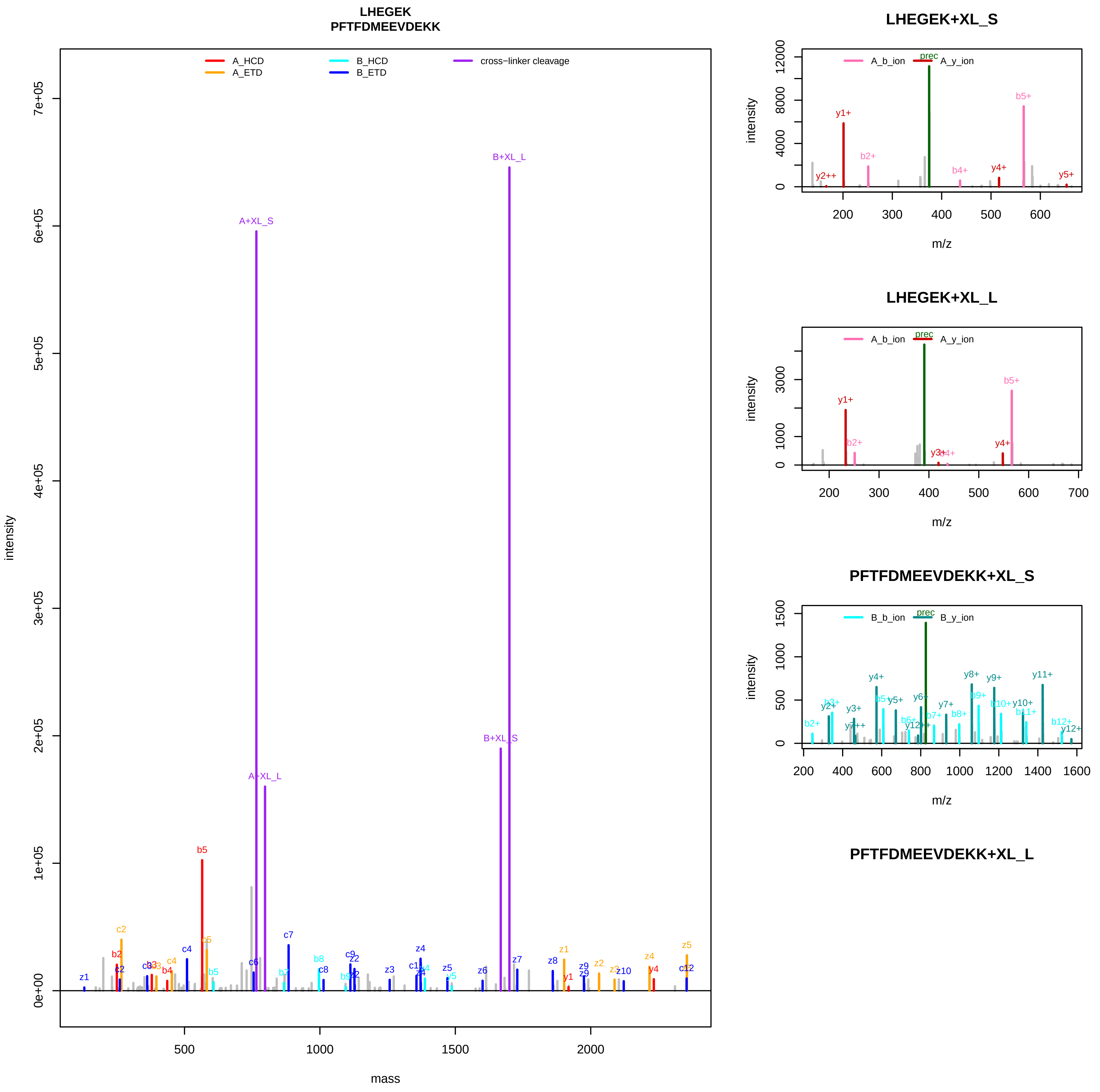
LHEGEK
PFTFDMEEVDEKK
A_HCD
A_ETD
B_HCD
B_ETD
cross−linker cleavage
intensity
mass
0e+00
1e+05
2e+05
3e+05
4e+05
5e+05
6e+05
7e+05
500
1000
1500
2000
A+XL_S
B+XL_L
A+XL_L
B+XL_S
b5
c2
c7
LHEGEK+XL_S
A_b_ion
A_y_ion
prec
y1+
b2+
b4+
y4+
b5+
y5+
y2++
m/z
LHEGEK+XL_L
y3+
PFTFDMEEVDEKK+XL_S
B_b_ion
B_y_ion
y4+
y8+
y9+
y11+
PFTFDMEEVDEKK+XL_L
